# Linking Healthcare Quality and Knowledge Management in Traditional and Ethnomedicine-Oriented Hospitals: A Scoping Review and Implementation Framework

**DOI:** 10.7759/cureus.115408

**Published:** 2026-08-29

**Authors:** Xiao Du, Nuo Wang, Abudukadier Aihemaijiang, Lei Zhao

**Affiliations:** 1 Faculty of Business and Communications, INTI International University, Nilai, MYS; 2 Department of Healthcare Quality and Safety, Luoyang Orthopedic-Traumatological Hospital of Henan Province (Henan Provincial Orthopedic Hospital), Luoyang, CHN; 3 Department of Medical Affairs, Hotan Prefecture Uyghur Medicine Hospital, Hotan, CHN; 4 Department of Administrative Affairs, Luoyang Orthopedic-Traumatological Hospital of Henan Province (Henan Provincial Orthopedic Hospital), Luoyang, CHN

**Keywords:** cultural and linguistic responsiveness, ethnomedicine, healthcare quality, integrative medicine, knowledge management, learning health system, patient safety, traditional medicine

## Abstract

Traditional and ethnomedicine-oriented hospitals operate at the intersection of culturally embedded clinical knowledge, contemporary biomedical evidence, regulatory requirements, and patient expectations. Their quality challenges therefore extend beyond conventional compliance and include the preservation, validation, translation, and safe application of knowledge. This scoping review mapped how healthcare quality management and knowledge management have been conceptualized and operationalized in hospital and health service settings relevant to traditional, complementary, integrative, Indigenous, and ethnomedicine-based care, and developed an implementation framework for Uyghur medicine services. The review followed Joanna Briggs Institute guidance, the Preferred Reporting Items for Systematic Reviews and Meta-Analyses Extension for Scoping Reviews (PRISMA-ScR), and PRISMA Extension for Reporting Literature Searches in Systematic Reviews (PRISMA-S). The information source plan covered PubMed/MEDLINE, PubMed Central, Scopus, Web of Science Core Collection, ProQuest, and Google Scholar, supplemented by the World Health Organization, International Organization for Standardization, Chinese policy and standards sources, publisher records, and citation chaining through July 27, 2026. Records were captured through directly accessible interfaces, database-targeted discovery, verified publisher metadata, and citation indexing. Ninety-six candidate records were captured; 73 unique records were screened, 60 full-text articles or complete source records were assessed, and 53 sources were included. Evidence clustered into seven interdependent domains: governance and accountability; evidence generation and context-sensitive standardization; preservation and transfer of tacit knowledge; patient safety and clinical risk; culturally and linguistically responsive person-centered care; digital knowledge infrastructure and organizational learning; and integration, referral coordination, and outcome measurement. Forty-one included sources were peer-reviewed publications, and 12 were authoritative policy or standards documents. Six sources directly addressed Uyghur medicine or immediately applicable ethnic-medicine policy and guidance, but none evaluated a hospital-wide quality management and knowledge management system in a Uyghur medicine hospital. We propose a Quality-Knowledge Integration Cycle linking traditional knowledge, external evidence, routine data, and patient experience to validation, codification, sharing, clinical application, and feedback against recognized quality dimensions. The framework offers a practical governance and research agenda for ethnomedicine-oriented hospitals while preserving epistemic distinctiveness and strengthening safety, equity, and learning.

## Introduction and background

Quality of care is commonly defined as the degree to which health services increase the likelihood of desired health outcomes and are consistent with current professional knowledge. The World Health Organization (WHO) describes high-quality services as effective, safe, people-centered, timely, equitable, integrated, and efficient [1-3]. These dimensions require more than accreditation or inspection. They depend on governance, a competent workforce, reliable information systems, medicines and technologies that are appropriately regulated, and organizational mechanisms that convert experience and evidence into better care [1-3].

Traditional, complementary, and integrative medicine has moved from the periphery of health policy toward explicit health system integration. For clarity, these terms are used as related but non-interchangeable descriptors in this review. Traditional medicine refers broadly to culturally rooted systems of medical knowledge and practice; complementary medicine refers to non-mainstream practices used alongside conventional care; integrative medicine refers to coordinated care that brings conventional and traditional or complementary approaches together; Indigenous medicine refers to health knowledge and practices grounded in Indigenous communities; and ethnomedicine-oriented care is used here to describe health services organized around a culturally distinctive medical tradition. The WHO Global Traditional Medicine Strategy 2025-2034 emphasizes four linked objectives: strengthening evidence, ensuring safety and regulation, integrating appropriate traditional and complementary practices into health systems, and optimizing their wider value [2]. A recent WHO-coordinated priority-setting exercise likewise identified patient safety, standardization, research capacity, and health system integration as central global priorities [4]. This policy direction creates a dual responsibility for traditional medicine hospitals. They must demonstrate recognizable standards of safety, effectiveness, equity, and accountability while retaining the diagnostic reasoning, therapeutic techniques, materia medica knowledge, and cultural meanings that define their medical tradition.

Uyghur medicine is a traditional medical system practiced mainly in Xinjiang, China. Published English-language literature describes its humoral foundations, diverse intellectual influences, distinctive formulations and practices, and ongoing modernization [5-7]. Most indexed research, however, concerns historical development, pharmacology, medicinal products, or clinical efficacy. Far less attention has been paid to the organizational systems through which Uyghur medical knowledge is governed, transmitted, standardized, audited, and linked to patient safety and service quality. This imbalance is important because the quality of a traditional medicine hospital cannot be inferred solely from the pharmacological quality of its products or the efficacy of selected interventions.

Knowledge management provides a complementary organizational lens. In healthcare, knowledge management includes the systematic creation, capture, appraisal, codification, sharing, retrieval, and application of knowledge to support decisions and improve performance [8-13]. It includes explicit knowledge, such as guidelines, pathways, formularies, electronic records, and indicator definitions, as well as tacit knowledge embodied in expert judgement, manual skills, pattern recognition, and culturally situated communication. Reviews consistently identify organizational culture, leadership, information technology, staff participation, trust, and opportunities for learning as core enablers [8-13]. Knowledge management is therefore not synonymous with digitization; it is the social and technical infrastructure that allows an organization to learn.

The intersection of healthcare quality and knowledge management is particularly consequential in ethnomedicine-oriented hospitals. Over-standardization can erase clinically meaningful distinctions and reduce traditional knowledge to simplified labels. Under-standardization can produce unwarranted variation, weak documentation, medication risk, and limited evaluability. Language discordance and cultural mismatch may further undermine informed consent, medication reconciliation, pain assessment, discharge education, incident reporting, and patient experience [14-20]. A balanced model must therefore standardize safety-critical processes while protecting legitimate pluralism in diagnosis and treatment.

Existing reviews address healthcare knowledge management, continuous quality improvement, hospital accreditation, cultural safety, language concordance, learning health systems, and traditional medicine integration [8-13,21-29], but these studies remain largely disconnected. Emerging governance work has begun to map the regulatory and health-system dimensions of traditional, complementary, and integrative medicine [30], while national and regional standards demonstrate that traditional medicine services can be linked to accreditation, information systems, workforce regulation, and quality monitoring [31-35]. However, no review identified by the authors has specifically mapped how healthcare quality management and knowledge management intersect in traditional and ethnomedicine-oriented hospital care. The primary objective of this scoping review was therefore to map the organizational concepts, mechanisms, practices, outcomes, and evidence gaps at this intersection. A secondary objective was to translate the findings into a context-sensitive implementation framework for Uyghur medicine services.

## Review

Methodology

Design and Reporting

This scoping review was designed using the framework proposed by Arksey and O'Malley, refined by Levac et al., and operationalized through updated Joanna Briggs Institute (JBI) guidance [36-39]. Reporting was guided by the Preferred Reporting Items for Systematic Reviews and Meta-Analyses Extension for Scoping Reviews (PRISMA-ScR) and, for the literature-search component, by the PRISMA Extension for Reporting Literature Searches in Systematic Reviews (PRISMA-S) [38,40]. The review protocol was not prospectively registered. The purpose was to map heterogeneous concepts, evidence types, and implementation mechanisms rather than estimate a pooled effect. Accordingly, no formal critical appraisal or risk-of-bias assessment of individual sources was performed. Methodological limitations were considered qualitatively during charting and synthesis to calibrate interpretation and transferability, rather than to generate quality scores or determine eligibility.

Eligibility Criteria

The prespecified eligibility criteria were structured according to the Population-Concept-Context (PCC) framework and are summarized in Table 1.

**Table 1:** Eligibility criteria. Table Credits: Xiao Du, Nuo Wang, Abudukadier Aihemaijiang, and Lei Zhao. This table was developed from the prespecified Population-Concept-Context eligibility framework applied in this scoping review. It is an original table and is not reproduced or adapted from any previously published table.

Element	Operational Definition
Population	Hospitals, hospital departments, health systems, managers, clinicians, patients, educators, or community partners involved in traditional, complementary, integrative, Indigenous, ethnic minority, or ethnomedicine-oriented care.
Concept	Healthcare quality management, clinical governance, patient safety, accreditation, quality improvement, outcome measurement, knowledge management, organizational learning, knowledge translation, knowledge preservation, standardization, digital knowledge systems, or intersections among these concepts.
Context	Hospital or health service settings globally. Evidence with direct relevance to traditional medicine services, culturally distinctive care, language-diverse populations, or China was prioritized during synthesis.
Evidence Types	Quantitative, qualitative, mixed-methods, implementation, quality improvement, systematic or scoping reviews, policy analyses, standards, guidelines, and authoritative institutional sources containing substantive organizational evidence.
Exclusions	Laboratory or pharmacology studies without an organizational quality or knowledge management component; efficacy trials without service delivery implications; purely commercial knowledge management systems; non-healthcare settings; sources with insufficient substantive information.
Languages	English and Chinese. Uyghur-language sources were eligible when a verified translation was available; none meeting the organizational eligibility criteria was identified in the completed public source search.

Information Sources

All bibliographic database searches and supplementary targeted searches were last updated on July 27, 2026. Database searches covered the period from database inception through that date. The information source plan comprised PubMed/MEDLINE, PubMed Central, Scopus, Web of Science Core Collection, ProQuest, and Google Scholar. The database-specific core search strings and supplementary targeted search approaches used in the review are summarized in Table 2. PubMed/MEDLINE and PubMed Central were searched directly. To extend coverage beyond biomedicine, records indexed or discoverable through Scopus, Web of Science, and ProQuest were sought using database-specific query translation, public discovery layers, verified publisher metadata, digital object identifier (DOI)/PubMed identifier (PMID) records, and citation indexes. Google Scholar was used for supplementary discovery and cited-by searching. As independently auditable platform-specific totals were not uniformly available for subscription-only databases, the PRISMA flow reports all candidate records actually captured and screened. Searches were supplemented by targeted searches of the WHO, the International Organization for Standardization (ISO), the National Health Commission of China, the National Administration of Traditional Chinese Medicine, the Xinjiang Uyghur Autonomous Region government, the Thai Department of Traditional and Alternative Medicine, relevant publisher websites, and the reference lists of key reviews.

**Table 2:** Database-specific core search strings and supplementary search strategies. Table Credits: Xiao Du, Nuo Wang, Abudukadier Aihemaijiang, and Lei Zhao. This table was developed specifically for this review and presents the database-specific search syntax and supplementary targeted-search approaches used to identify eligible sources. It is an original table and is not reproduced or adapted from any previously published table. MEDLINE: Medical Literature Analysis and Retrieval System Online; WHO: World Health Organization; ISO: International Organization for Standardization; tiab: title and abstract; TITLE-ABS-KEY: title, abstract, and keywords; TS: topic search; NOFT: anywhere except full text.

Source	Core Reproducible Search String
PubMed/MEDLINE and PubMed Central	(("traditional medicine"[tiab] OR "complementary medicine"[tiab] OR "integrative medicine"[tiab] OR ethnomedicine[tiab] OR "indigenous medicine"[tiab]) AND (hospital*[tiab] OR "health service*"[tiab]) AND ("quality management"[tiab] OR "quality improvement"[tiab] OR "patient safety"[tiab] OR "clinical governance"[tiab] OR accreditation[tiab] OR standardization[tiab])) OR (("knowledge management"[tiab] OR "organizational learning"[tiab] OR "knowledge translation"[tiab] OR "learning health system*"[tiab]) AND (hospital*[tiab] OR healthcare[tiab] OR "health service*"[tiab]) AND (quality[tiab] OR safety[tiab] OR improvement[tiab]))
Scopus	TITLE-ABS-KEY(("traditional medicine" OR "complementary medicine" OR "integrative medicine" OR ethnomedicine OR "indigenous medicine") AND (hospital* OR "health service*") AND ("quality management" OR "quality improvement" OR "patient safety" OR "clinical governance" OR accreditation OR standardization OR "knowledge management" OR "organizational learning" OR "learning health system*"))
Web of Science Core Collection	TS=(("traditional medicine" OR "complementary medicine" OR "integrative medicine" OR ethnomedicine OR "indigenous medicine") AND (hospital* OR "health service*") AND ("quality management" OR "quality improvement" OR "patient safety" OR "clinical governance" OR accreditation OR standardization OR "knowledge management" OR "organizational learning" OR "learning health system*"))
ProQuest	NOFT(("traditional medicine" OR "complementary medicine" OR "integrative medicine" OR ethnomedicine OR "indigenous medicine") AND (hospital* OR "health service*") AND ("quality management" OR "quality improvement" OR "patient safety" OR "clinical governance" OR accreditation OR standardization OR "knowledge management" OR "organizational learning" OR "learning health system*"))
Google Scholar	Short-form searches were run separately because of platform syntax limits: (1) "traditional medicine hospital" quality safety governance; (2) ethnomedicine hospital "knowledge management"; (3) "indigenous health services" cultural safety quality; (4) "Uyghur medicine" OR "Uighur medicine" hospital quality management. Cited-by searching was performed for sentinel reviews and standards.
Language and Cultural Responsiveness	("language concordance" OR "language discordance" OR "language barrier*" OR "cultural safety" OR "culturally responsive") AND (hospital* OR healthcare OR "health service*") AND (quality OR safety OR outcome*)
Uyghur/Uighur and Chinese-Language Targeted Search	Targeted searches combined "Uyghur medicine" and "Uighur medicine" with English-language terms related to hospitals, healthcare quality, patient safety, guidelines, standards, accreditation, management, and knowledge management. Parallel Chinese-language searches used equivalent terms for Uyghur medicine, ethnic medicine, hospitals, healthcare quality, patient safety, guidelines, standards, accreditation, management, and knowledge management.
Authoritative Policy and Standards	Targeted WHO, ISO, Chinese national and Xinjiang policy, National Administration of Traditional Chinese Medicine, and Thai traditional-medicine searches for quality, safety, accreditation, integration, information systems, workforce, and medical-quality governance.
Citation Chaining	Backward reference list screening and forward cited-by searching of key knowledge management, quality management, cultural safety, traditional medicine governance, and Uyghur medicine sources.

Search Strategy

The overall search approach covered six complementary streams: (1) traditional, complementary, integrative, Indigenous, and ethnomedicine services combined with healthcare quality or patient safety; (2) healthcare knowledge management, organizational learning, and learning health systems; (3) language concordance, language barriers, cultural safety, and culturally responsive care; (4) Uyghur/Uighur medicine using English and Chinese terms; (5) authoritative policy, regulation, accreditation, and standards; and (6) backward reference-list screening and forward cited-by searching. Database searches covered the period from database inception to July 27, 2026. Both the preferred spelling "Uyghur" and the historical spelling "Uighur" were searched. Chinese-language searches used equivalent terms for Uyghur medicine, ethnic medicine, healthcare quality, patient safety, knowledge management, knowledge translation, organizational learning, hospital management, and hospital accreditation. These unrestricted inception-to-date searches, together with targeted Chinese-language and Uyghur/Uighur searches, were intended to maximize capture of both recent literature through 2026 and context-specific evidence relevant to ethnic-medicine services.

Study Selection

Candidate records were entered into a structured screening workbook and deduplicated by normalized title, DOI, or PMID, and manual comparison. Two reviewers (X.D. and N.W.) independently assessed records against the prespecified PCC eligibility criteria. X.D. applied a sensitivity-oriented assessment that retained potentially transferable organizational evidence, whereas N.W. applied a specificity-oriented assessment requiring an explicit healthcare quality, patient safety, service delivery, governance, or knowledge management mechanism. Any record retained by either reviewer proceeded to source-level assessment. Eligibility disagreements were adjudicated by the third reviewer (A.A.) after reapplication of the eligibility criteria to the complete abstract, full text, or authoritative source.

Data Charting

Following study selection, data were charted using a piloted structured form that captured publication year, country or region, medical tradition, setting, design, quality domain, knowledge process, organizational mechanism, outcomes or indicators, barriers, facilitators, and relevance to Uyghur medicine services. The charted fields and operational categories are presented in the Appendices. The review retained an audit trail of duplicate status, screening decisions, reasons for exclusion, and charted information.

Synthesis

Descriptive characteristics of the included evidence were summarized as counts using mutually exclusive primary design classifications. No meta-analysis or inferential statistical analysis was planned or performed; all numerical summaries were descriptive. Thematic synthesis combined a deductive coding structure based on WHO quality dimensions and knowledge management processes with inductive coding of recurrent organizational mechanisms. Each source was assigned a primary evidence classification according to its dominant contribution for descriptive reporting, while thematic overlap was retained during interpretation. Themes were compared across conventional hospitals, traditional medicine institutions, integrative services, and culturally or linguistically distinctive care settings. Transferability to Uyghur medicine was assessed at the level of organizational mechanism rather than medical-system identity. Evidence was considered potentially transferable when it addressed a comparable organizational problem, such as tacit knowledge loss, bilingual communication, medication safety, pathway standardization, incident learning, or integration between medical systems. Implementation-oriented propositions were generated only when at least two evidence streams converged or when an authoritative standard directly addressed the relevant mechanism.

Results

Search Results and Characteristics of the Evidence

**Figure 1:**
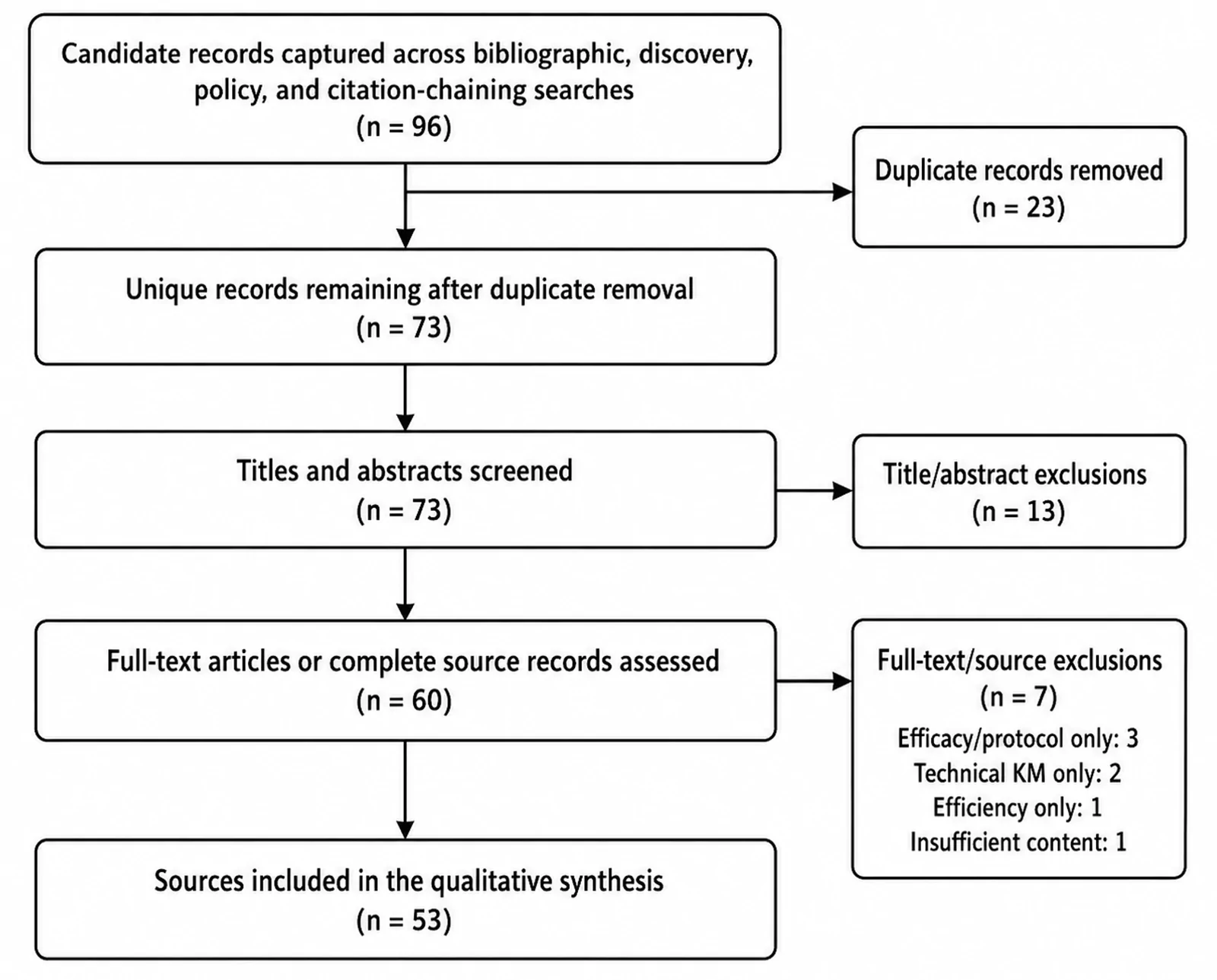
PRISMA-ScR flow of source identification and selection. Of 96 candidate records, 23 duplicates were removed, leaving 73 records for title/abstract screening. After 13 title/abstract exclusions and seven full-text/source exclusions, 53 sources were included in the qualitative synthesis. PRISMA-ScR: Preferred Reporting Items for Systematic Reviews and Meta-Analyses Extension for Scoping Reviews; KM: knowledge management.

The expanded search and discovery process captured 96 candidate records. Twenty-three duplicates were removed, leaving 73 unique records for title and abstract screening. Thirteen records were excluded at this stage because they were laboratory, pharmacokinetic, preclinical, purely technical, or otherwise outside the healthcare service context. Sixty full-text articles or complete authoritative sources were assessed, and seven were excluded, including three efficacy studies or protocols without organizational implications, two technical knowledge extraction or model development studies outside hospital quality management, one hospital efficiency study without substantive quality or knowledge management outcomes, and one source with insufficient organizational content. Fifty-three sources were included in the qualitative synthesis. The source identification, screening, eligibility assessment, and inclusion process is summarized in Figure 1. Of these, 41 were peer-reviewed journal publications, and 12 were authoritative policy, regulatory, accreditation, or standards documents. Publication years ranged from 2007 to 2026. Using a mutually exclusive primary classification, the corpus comprised 21 reviews or evidence syntheses, eight organizational empirical or priority-setting studies, four patient safety audits or record review studies, eight conceptual or policy analyses, and 12 authoritative policy or standards sources. Six sources directly concerned Uyghur medicine or provided immediately applicable ethnic-medicine policy or accreditation guidance [5-7,32,33,41], but none evaluated a hospital-wide system integrating healthcare quality management and knowledge management in a Uyghur medicine hospital.

Characteristics of Included Sources

The principal characteristics of the included evidence base, including publication type, publication period, primary design classification, direct relevance to Uyghur medicine, and geographical breadth, are summarized in Table 3.

**Table 3:** Characteristics of included sources. Table Credits: Xiao Du, Nuo Wang, Abudukadier Aihemaijiang, and Lei Zhao. This table summarizes the characteristics of the 53 sources included in the qualitative synthesis based on the review’s prespecified screening and data-charting framework. Source categories were assigned using mutually exclusive primary classifications for descriptive purposes. This table is original and is not reproduced or adapted from any previously published table.

Characteristic	Result
Included Sources	53
Publication Type	41 peer-reviewed publications; 12 authoritative policy, regulatory, accreditation, or standards sources
Publication Period	2007-2026
Primary Design Classification	21 reviews/evidence syntheses; 8 organizational empirical or priority-setting studies; 4 safety audits/record reviews; 8 conceptual/policy analyses; 12 policy/standards sources
Direct Uyghur Medicine Evidence	Six direct or immediately applicable context, guideline, policy, or accreditation sources; no hospital-wide quality-plus-knowledge-management evaluation
Geographical Breadth	Global and multinational evidence plus studies from China, Japan, South Korea, Europe, Canada, Australia, and other health system contexts

Evidence Map

To facilitate thematic interpretation across the heterogeneous evidence base, the included sources were organized into major evidence clusters according to their principal contribution to healthcare quality, knowledge management, traditional medicine governance, patient safety, cultural and linguistic responsiveness, learning systems, and care integration. The resulting evidence map is presented in Table 4.

**Table 4:** Evidence map of healthcare quality and knowledge management in traditional and ethnomedicine-oriented hospital care. Table Credits: Xiao Du, Nuo Wang, Abudukadier Aihemaijiang, and Lei Zhao. This table represents an original synthesis of the included peer-reviewed literature and authoritative policy, regulatory, accreditation, and standards sources. Evidence was organized into thematic clusters based on recurrent organizational mechanisms, consistent findings, and their potential implications for Uyghur medicine services. Transferability was assessed at the level of organizational mechanisms rather than assumed equivalence between different medical traditions. Citation numbers correspond to the reference list and identify representative sources supporting each evidence cluster. WHO: World Health Organization; ISO: International Organization for Standardization; TCIM: traditional, complementary, and integrative medicine; KM: knowledge management; TCM: traditional Chinese medicine.

Evidence Cluster	Representative Literature	Consistent Finding	Implication for Uyghur Medicine Services
Healthcare Quality	WHO quality frameworks; Kruk et al.; accreditation reviews; ISO 7101; continuous quality improvement review [1-3,24-26,42-46]	Quality is multidimensional, and accreditation or certification is most useful when linked to measurement, feedback, and continual improvement.	Use accreditation as the governance floor, with pathway-level outcome and learning systems above it.
Traditional Medicine Governance	WHO 2025-2034 strategy; integration reviews; TCIM governance framework; Thai service and accreditation guidance [2,4,30,31,47-49]	Evidence, regulation, safety, information systems, workforce, financing, and integration must advance together.	Link preservation of Uyghur medicine to explicit safety, evidence, integration, and outcome systems.
Uyghur Medicine	Historical and modernization reviews; ethics of integration; guideline appraisal; ethnic-medicine accreditation and Xinjiang policy [5-7,32-34,41]	A distinct knowledge base and explicit policy framework are documented, but direct organizational quality research remains sparse, and guideline rigor/applicability require strengthening.	Establish a service research program on governance, safety, knowledge transfer, and outcomes, not only products and efficacy.
Knowledge Management	Healthcare KM reviews; Chinese hospital study; European qualitative study; recent systematic reviews [8-13,27,28,50,51]	Leadership, culture, trust, staff participation, boundary spanning, and useful technology enable capture and use of knowledge.	Treat KM as a clinical governance function jointly led by medical, quality, nursing, pharmacy, information, and education teams.
Learning Health Systems	Competency and evidence-based practice reviews; collective learning literature	Routine data improve care only when converted into credible knowledge, returned to teams, and linked to implementation capability.	Build data-to-knowledge, knowledge-to-performance, and performance-to-data cycles.
Patient Safety	TCM safety culture survey; Korean traditional medicine record review; Kampo incident analysis	Safety culture, event reporting, multidisciplinary review, and tradition-specific risk categories are necessary.	Create non-punitive incident learning and pharmacovigilance covering conventional and traditional therapies.
Language and Cultural Responsiveness	Language concordance reviews; hospital safety study; cultural safety syntheses and definition work [14-20,29]	Communication discordance can affect diagnosis, medication, consent, trust, access, and outcomes; training alone is insufficient.	Make bilingual communication quality measurable across consent, medication, pain, discharge, complaints, and patient-reported experience.
Integration and Coordination	Traditional medicine integration reviews; TCIM governance; Thai integrative service standards; culturally responsive service models [20,30,31,47-49,52]	Integration succeeds when governance, roles, referral, information exchange, workforce, financing, and community participation are explicit.	Define interfaces between Uyghur medicine, biomedicine, emergency care, diagnostics, pharmacy, and referral networks.

Theme 1: Governance Must Join Quality Accountability With Knowledge Stewardship

Across the literature, quality and knowledge activities were frequently managed in parallel. Quality teams focused on standards, incidents, indicators, and accreditation, while education, research, information technology, pharmacy, and senior clinicians managed different knowledge assets. Systematic reviews and hospital studies consistently identified leadership, organizational structure, culture, trust, staff participation, information infrastructure, knowledge acquisition, knowledge sharing, knowledge application, and knowledge storage as determinants of knowledge creation and use [8-13,27,28,50,51]. Quality improvement evidence similarly emphasized leadership, resources, professional engagement, measurement, feedback, and sustained improvement capability [1-3,24-26,42-44,46,53].

Theme 2: Evidence Generation and Standardization Must Avoid Epistemic Erasure

Traditional medicine integration creates a tension between comparability and fidelity. WHO policy and recent global priority-setting support evidence generation, safety monitoring, standardized terminology, regulation, research capacity, and appropriate health system integration [2,4,47-49,52]. Comparative governance work shows that traditional medicine is shaped by interacting rules for jurisdiction, workforce, service delivery, information management, financing, knowledge protection, and intersectoral accountability [30]. National and regional examples demonstrate that traditional medicine services can be linked to hospital accreditation, service standards, licensing, information systems, and quality monitoring [31-35]. At the same time, critical scholarship warns that applying biomedical standards without examining their assumptions may marginalize legitimate traditional concepts or alter the intervention being evaluated [7]. The guideline appraisal by Deng et al., which included 10 Uyghur medicine guidelines within a set of 75 traditional medicine guidelines, demonstrates that standardization alone does not assure methodological rigor or applicability [41].

Theme 3: Tacit Knowledge Preservation Is a Patient Safety Function

Ethnomedicine relies heavily on tacit knowledge, which involves sensory examination, pattern recognition, preparation techniques, manual skills, contextual judgement, and culturally meaningful explanation. These capabilities are vulnerable to retirement, staff turnover, informal apprenticeship, and inconsistent documentation. Knowledge management literature distinguishes codification strategies, which place explicit knowledge in repositories, from personalization strategies, which connect staff with experienced practitioners [8-13]. Neither is sufficient alone. Video-supported procedure libraries, structured case conferences, supervised competency portfolios, communities of practice, bilingual terminology glossaries, and expert succession plans can make knowledge accessible without pretending that all expertise can be reduced to text.

Theme 4: Patient Safety Requires Tradition-Specific Surveillance and a Just Culture

Patient safety culture evidence consistently identifies teamwork, communication openness, staffing, leadership support, non-punitive response, and feedback after reporting as core determinants of learning [23,54,55]. In four traditional Chinese medicine institutions, Zhang et al. obtained 522 valid responses; communication about error scored highest, whereas reporting of patient safety events scored lowest [54]. Direct event data reinforce the need for tradition-specific surveillance. In two Korean traditional medicine teaching hospitals, 122 of 1,152 reviewed admissions (10.6%) had at least one adverse event, 70.7% of events were judged preventable, and procedure-related events were most common [56]. A 10-year Kampo incident analysis likewise identified medication errors and adverse drug events requiring product- and workflow-specific prevention [57]. More broadly, collective-learning and system-level patient safety frameworks emphasize organizational learning, feedback, and prevention rather than isolated individual blame [58,59].

Theme 5: Cultural and Linguistic Responsiveness Is a Core Quality Mechanism

Language and culture are sometimes framed as patient experience issues, yet the evidence links communication barriers with risks in medication administration, pain management, diagnosis, risk communication, discharge, and acute care [14-17]. Reviews of language concordance show generally favorable associations with interpersonal processes and some health outcomes, although study designs remain heterogeneous [15-17]. Cultural safety research emphasizes two-way communication, trust, an adequately resourced culturally representative workforce, responsive systems, meaningful community influence, and organizational accountability for care as experienced by patients and communities [18-20,29].

Theme 6: Digital Infrastructure Must Support a Learning Health System, Not Merely Data Storage

Electronic records and repositories are valuable only when they support retrieval, decision-making, feedback, and practice change. Learning health system literature describes a cycle in which internal data and experience are integrated with external evidence, converted into knowledge, applied in care, and evaluated continuously [21-23]. Ethnomedicine documentation presents additional challenges: traditional diagnoses may not map cleanly to biomedical terminologies; free text may contain essential reasoning; and bilingual terms may be inconsistent.

Theme 7: Integration Requires Explicit Interfaces, Referral Rules, and Shared Outcomes

Integration is not achieved by co-locating different medical traditions. Reviews of traditional medicine integration, comparative governance frameworks, national service models, culturally responsive care, and the WHO framework on integrated, people-centered health services identify governance, professional roles, referral pathways, financing, information exchange, workforce regulation, community participation, and mutual respect as recurring determinants [20,30,31,47-49,52,60].

Author-derived implementation synthesis

The following Quality-Knowledge Integration Cycle, operational framework, and phased implementation roadmap are author-derived synthesis outputs informed by the included evidence. They are presented as mechanism-informed proposals rather than direct empirical findings from individual included sources and require prospective validation in local implementation settings.

The Quality-Knowledge Integration Cycle

**Figure 2:**
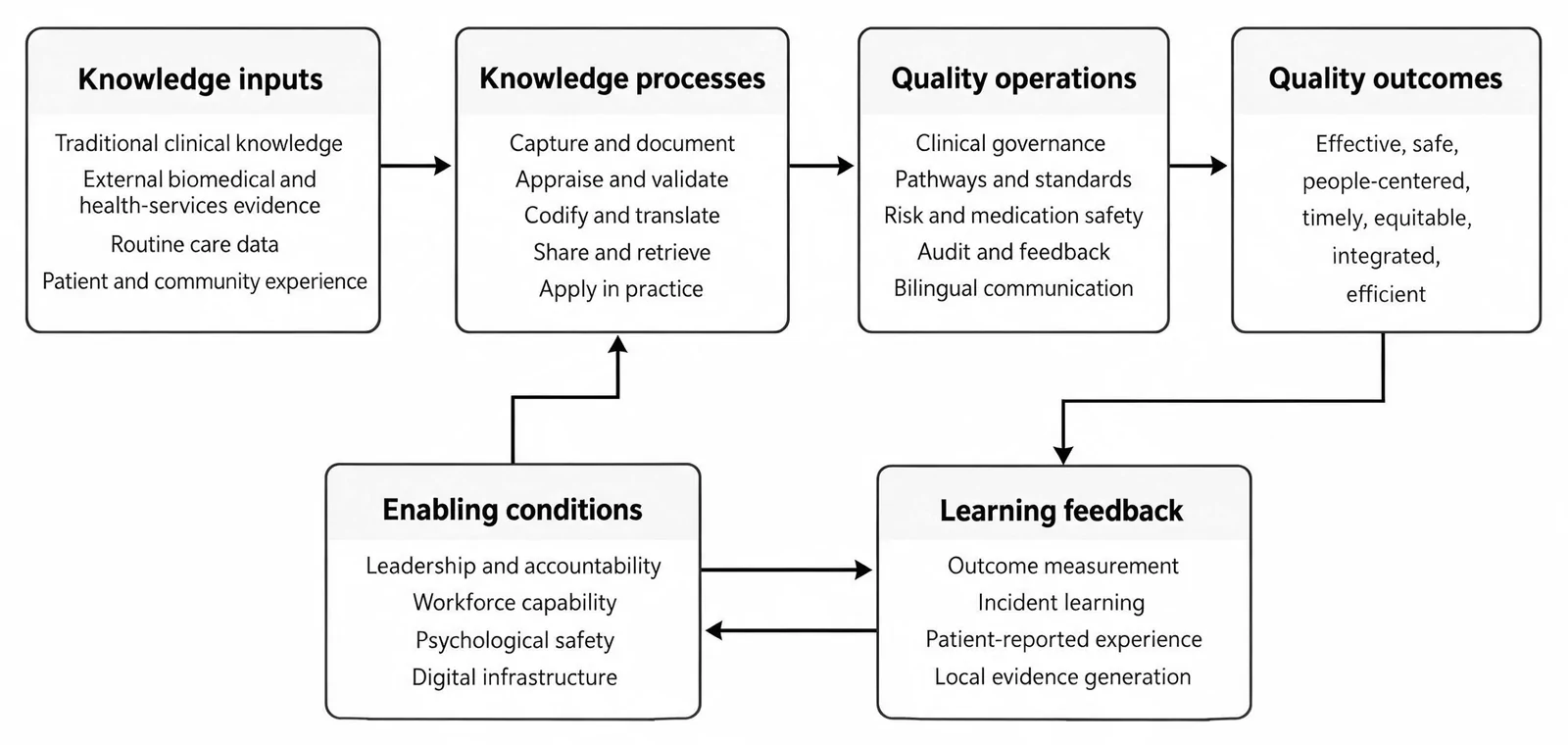
Quality-Knowledge Integration Cycle for ethnomedicine-oriented hospitals. Original figure created with PowerPoint software (Microsoft Corporation, Redmond, WA). The model was developed from the thematic synthesis of the included evidence and illustrates how traditional clinical knowledge, external biomedical and health-services evidence, routine care data, and patient and community experience are captured, appraised, translated, shared, and applied through organizational knowledge processes. These processes inform clinical governance, pathways and standards, risk and medication safety, audit and feedback, and bilingual communication, with performance assessed against recognized quality dimensions. Outcome measurement, incident learning, patient-reported experience, and local evidence generation provide learning feedback, while leadership and accountability, workforce capability, psychological safety, and digital infrastructure function as enabling conditions for the cycle. The framework represents a mechanism-informed synthesis and should be prospectively evaluated in local implementation settings.

The synthesis supports a Quality-Knowledge Integration Cycle that integrates knowledge inputs, knowledge processes, quality operations, enabling conditions, and learning feedback into a continuous organizational learning system for ethnomedicine-oriented hospitals (Figure 2). Four knowledge inputs that include traditional clinical knowledge, external biomedical and health services evidence, routine clinical and operational data, and patient/community experience enter a set of knowledge processes, comprising capture, appraisal, codification or translation, sharing, and application. These processes feed quality operations such as clinical governance, pathways, credentialing, risk management, medication safety, audit and feedback, and culturally responsive communication. Outcomes are assessed against the seven WHO quality dimensions. The results return through incident learning, outcome measurement, and patient feedback to refine the knowledge base. Leadership, workforce capability, psychological safety, and digital infrastructure enable the full cycle.

Proposed Operational Framework for Uyghur Medicine Hospitals

To translate the Quality-Knowledge Integration Cycle into actionable hospital-level components, the synthesis was operationalized into eight domains with corresponding core mechanisms and example indicators, as shown in Table 5.

**Table 5:** Proposed operational framework for quality-knowledge integration in Uyghur medicine hospitals. Table Credits: Xiao Du, Nuo Wang, Abudukadier Aihemaijiang, and Lei Zhao. This table represents an original operational framework developed from the evidence synthesis presented in this review and the proposed Quality-Knowledge Integration Cycle. The domains, core mechanisms, and example indicators were formulated by the authors to translate the synthesized evidence into potentially actionable components for Uyghur medicine hospitals and are not reproduced or adapted from any single previously published framework or table. The example indicators are illustrative and require local validation before implementation. PROMs: patient-reported outcome measures; PREMs: patient-reported experience measures.

Domain	Core Mechanisms	Example Indicators
1. Governance and Accountability	Joint quality and knowledge council; named executive sponsor; patient/community representation	Board review of quality-knowledge dashboard; action closure rate; policy version control
2. Knowledge Inventory and Preservation	Map clinical pathways, expert skills, formulations, procedures, terminology, and high-risk knowledge gaps	Proportion of priority services with current knowledge assets; succession coverage; competency portfolio completion
3. Evidence and Standardization	Three-tier classification: universal safety standards, tradition-specific consensus standards, research-governed practices	Pathway coverage; adherence with justified exceptions; evidence update interval; registry enrollment
4. Patient Safety and Pharmacovigilance	Tradition-specific event taxonomy; non-punitive reporting; multidisciplinary review; product traceability	Reporting rate; serious event review timeliness; recurrence; batch traceability; herb-drug interaction screening
5. Bilingual, Culturally Responsive Care	Record language preference; interpreter standards; bilingual consent, education, and complaint analysis	Language documentation; teach-back completion; comprehension; PREMs; disparity measures
6. Digital Learning Infrastructure	Bilingual data dictionary; structured-plus-narrative record; searchable repository; decision-oriented dashboards	Data completeness; retrieval/use metrics; indicator timeliness; number of audit-feedback cycles
7. Integrated Pathways and Outcomes	Referral/escalation protocols; multidisciplinary case review; shared outcome sets	Referral completion; diagnostic delay; PROMs/PREMs; readmission; functional outcomes; cost/resource use
8. Research and External Learning	Prospective registries; implementation studies; multicenter collaboration; publication and guideline feedback	Registry completeness; studies initiated; evidence-to-policy updates; dissemination and adoption

A Phased Implementation Roadmap for the Hotan Context

Table 6 presents an illustrative, author-derived phased implementation roadmap for the Hotan context. It is intended as a prospective implementation proposal rather than an empirically validated sequence and should be adapted using local institutional data, resources, priorities, and stakeholder input.

**Table 6:** Proposed phased implementation roadmap for the Hotan context. Table Credits: Xiao Du, Nuo Wang, Abudukadier Aihemaijiang, and Lei Zhao. This table presents an original, context-sensitive implementation roadmap developed by the authors to translate the proposed operational framework into a feasible sequence for the Hotan context. The phases, priority actions, outputs, and suggested time frames are implementation proposals informed by the synthesis in this review rather than empirically validated implementation intervals. They should be adapted and prospectively evaluated using local institutional data, resources, priorities, and stakeholder input. This table is not reproduced or adapted from any previously published table. PDSA: Plan-Do-Study-Act; WHO: World Health Organization.

Phase	Priority Actions	Outputs
0-3 Months: Establish the Baseline	Create a joint quality-knowledge steering group; inventory priority services and knowledge assets; map current indicators, incident systems, bilingual processes, and referral interfaces; select two high-volume or high-risk pilot pathways.	Baseline map, governance terms of reference, gap register, agreed indicator dictionary.
4-9 Months: Codify and Pilot	Develop bilingual minimum documentation, safety checklists, competency criteria, expert-consensus pathways, and tradition-specific incident categories; train pilot teams and use rapid PDSA cycles.	Pilot adherence, documentation completeness, staff feedback, incident learning, patient comprehension.
10-18 Months: Build the Learning Cycle	Integrate structured fields into the electronic record; establish dashboards, monthly multidisciplinary learning reviews, patient-reported measures, and prospective registries.	Trend data across WHO quality domains; variation and equity analysis; completed improvement cycles.
18-36 Months: Evaluate and Scale	Use interrupted time-series, mixed-methods implementation studies, or stepped rollout designs; scale effective components; share bilingual tools; collaborate with other ethnic medicine institutions.	Clinical, safety, experience, workforce, and organizational-learning outcomes; peer-reviewed outputs.

Discussion

This review reframes knowledge management as an operating mechanism of healthcare quality in traditional and ethnomedicine-oriented hospitals. The central finding is not simply that quality management and knowledge management coexist, but that each is incomplete without the other. Quality systems require a defensible account of what the organization knows, how that knowledge is validated, who can use it, and how it changes after new evidence or adverse events. Knowledge preservation programs, in turn, cannot demonstrate value unless they are connected to clinical consistency, safety, patient experience, equity, and outcomes. The proposed Quality-Knowledge Integration Cycle makes this interdependence explicit and converts an abstract relationship into a governable sequence of inputs, knowledge processes, quality operations, outcomes, and feedback.

The evidence base is asymmetrical. Healthcare knowledge management, organizational learning, and continuous quality improvement literatures are relatively mature [8-13,21-28,50,51,53,58], whereas hospital-level quality research within traditional medicine is thinner, and direct organizational evidence for Uyghur medicine remains exceptionally sparse. The expanded search added explicit ethnic-medicine accreditation requirements and Xinjiang policy commitments to standardized construction, quality systems, safety, service capability, and distinctive knowledge preservation [32-34]. These sources clarify the policy environment but do not substitute for empirical evaluation. This broader governance context is also reflected in hospital-level medical quality evaluation in tertiary traditional Chinese medicine institutions and China's national requirements for medical quality management [61,62]. None examined how a Uyghur medicine hospital governs expert knowledge, monitors tradition-specific risk, measures bilingual communication, or closes audit-feedback cycles. This absence should not be interpreted as evidence that such mechanisms are unimportant or absent in practice. It indicates that they have not been sufficiently studied or reported in indexed literature, making service-level implementation research a high-value contribution.

A major interpretive issue is transferability. Evidence from conventional hospitals provides robust mechanisms for governance, incident learning, audit and feedback, and organizational capability, but it does not determine which elements of Uyghur clinical reasoning are clinically meaningful. Evidence from traditional Chinese medicine, Kampo, Korean traditional medicine, and integrative medicine is closer in organizational form, yet still cannot be assumed to represent Uyghur medicine. The appropriate unit of transfer is therefore the mechanism, not the medical identity. A non-punitive event review process, for example, is transferable; the event taxonomy and expert interpretation must be locally developed. Similarly, bilingual terminology governance is transferable as a process, whereas the vocabulary, diagnostic categories, and acceptable mappings require Uyghur medicine expertise.

The three-tier standardization model addresses a recurring paradox. Uniformity is indispensable for safety-critical processes, but excessive standardization can strip traditional practice of context and encourage superficial documentation. Conversely, unrestricted variation impairs traceability, competency assessment, and learning. The proposed distinction among universal safety standards, tradition-specific consensus standards, and research-governed practices offers a practical middle position. It also makes exceptions auditable when clinicians depart from a pathway, since the rationale, expected benefit, risk, and follow-up are documented. This shifts quality management from rigid conformity toward accountable variation.

Tacit knowledge should be treated as a patient safety asset rather than solely as cultural heritage. Sensory examination, pattern recognition, preparation techniques, manual skills, and culturally situated explanations are vulnerable to retirement, turnover, and informal apprenticeship. The knowledge management literature indicates that codification and personalization are complementary rather than competing strategies [8-13,42,43]. Video-supported procedure libraries, structured case conferences, supervised competency portfolios, communities of practice, bilingual terminology glossaries, and succession plans can preserve access to expertise, while direct supervision and assessment acknowledge that not all knowledge can be reduced to text. Linking these tools to credentialing, variation review, and outcomes makes preservation clinically accountable.

Cultural and linguistic responsiveness also emerges as structural quality infrastructure. Language discordance is associated with failures in medication communication, diagnosis, consent, discharge, trust, and continuity [14-17]. Cultural safety requires more than individual cultural competence. Organizations should address power, accountability, workforce representation, service design, and whether care is experienced as safe by patients and communities [18-20,29]. A multilingual hospital therefore needs to move beyond the presence of translated forms to a closed-loop standard, which consists of recording language preference, providing qualified language support, using teach-back for safety-critical information, documenting comprehension, and examining disparities in complaints, readmissions, adverse events, and patient-reported experience.

The digital implication is equally specific. Electronic records should preserve the original Uyghur medicine diagnosis and reasoning while supporting structured fields for symptoms, safety screening, intervention, preparation or product traceability, outcomes, and adverse events. A bilingual, version-controlled data dictionary is more important than forcing one-to-one mappings where conceptual equivalence does not exist. Dashboards should be decision-oriented, having the feature of identifying clinically important variation, delayed escalation, recurrent harm, inequity, missing documentation, and knowledge assets at risk. This design prevents digitization from becoming passive storage and makes it part of a learning health system.

Specifically, for a Uyghur medicine hospital, the framework is most defensibly implemented at pathway level before hospital-wide scale-up. Two pilots would generate publishable and operationally useful evidence. One is a bilingual medication safety and discharge-comprehension pathway, and another one is a high-volume Uyghur medicine clinical pathway linked to a prospective registry, expert consensus documentation, competency assessment, and patient-reported outcomes. A mixed-methods implementation design could measure adoption, fidelity with justified exceptions, staff acceptability, safety events, comprehension, functional outcomes, and variation across language groups. Interrupted time series analysis, phased rollout, or controlled before-after designs would be stronger than a one-time satisfaction survey.

This review also generates testable propositions. First, joint quality-and-knowledge governance should improve action closure and reduce unresolved variation compared with separated committees. Second, structured-plus-narrative bilingual documentation should improve traceability without materially reducing fidelity to traditional reasoning. Third, tradition-specific incident taxonomy and multidisciplinary review should increase meaningful reporting before reducing recurrence. Fourth, competency portfolios and succession planning should reduce unexplained practitioner variation. These propositions convert the framework from a conceptual diagram into a research program that can be evaluated, refined, and shared across ethnic medicine institutions.

Limitations

This review has several limitations. First, although the information source plan and reproducible syntax were extended to Scopus, Web of Science Core Collection, ProQuest, and Google Scholar, direct export and independently auditable platform-specific totals from subscription-only interfaces were not uniformly available in the review environment. Coverage was strengthened through database-specific query translation, public discovery layers, publisher metadata, DOI/PMID cross-checking, Google Scholar discovery, and citation chaining, but this is not equivalent to a librarian-executed institutional export from every platform. The PRISMA flow therefore reports records actually captured and screened rather than unverified database totals. Source-specific retrieval totals were not retrospectively reconstructed because contemporaneous auditable platform exports were unavailable for all discovery routes, and retrospective reconstruction would introduce unverifiable precision. Second, the review was not prospectively registered, as its primary purpose was to map broad, heterogeneous concepts, policy documents, and implementation mechanisms rather than to evaluate a single intervention or estimate pooled effect sizes. Third, terminology spans multiple disciplines, and relevant studies may not use the labels "quality management” or “knowledge management.” Fourth, direct Uyghur-language and hospital-level Uyghur medicine evidence was limited, so several recommendations rely on mechanism-based transfer from other traditional medicine, culturally responsive, and conventional hospital settings. Fifth, the included evidence was heterogeneous and often observational, and the proposed framework is a mechanism-informed synthesis rather than proof that every component improves patient outcomes. Finally, scoping reviews map rather than weight evidence, and local implementation should therefore be prospectively evaluated rather than assumed effective.

## Conclusions

Healthcare quality management and knowledge management are closely interdependent in traditional and ethnomedicine-oriented hospitals. Across the 53 included sources, recurrent priorities included integrated governance and accountability, context-sensitive standardization, preservation of tacit knowledge, tradition-specific patient safety, culturally and linguistically responsive care, learning-oriented digital infrastructure, and explicit mechanisms for integration, referral coordination, and outcome measurement. Evidence directly addressing Uyghur medicine remains limited; importantly, the absence of a comprehensively evaluated hospital-wide quality-and-knowledge-management model in the literature identified by this review should be interpreted as a gap in published organizational evidence rather than evidence that relevant practices are absent from existing institutions.

The proposed Quality-Knowledge Integration Cycle translates these findings into a context-sensitive framework linking traditional clinical expertise, external evidence, routine service data, and patient and community experience with knowledge processes, quality operations, and continuous organizational learning. Its practical value should now be prospectively evaluated through transparent, bilingual, pathway-level implementation studies in Uyghur medicine hospitals, with attention to feasibility, adoption, fidelity, safety, equity, patient experience, clinical outcomes, and organizational learning, while preserving the conceptual integrity and legitimate clinical distinctiveness of Uyghur medicine.

## Appendices

**Table 7:** PRISMA-ScR reporting checklist. The reporting of this scoping review was guided by PRISMA-ScR [38]. PRISMA-ScR: Preferred Reporting Items for Systematic Reviews and Meta-Analyses Extension for Scoping Reviews.

Section	Item	Reporting Requirement	Location in Manuscript
Title	1	Identification of the article as a scoping review	Title
Abstract	2	Structured summary of rationale, methods, sources, results, and conclusions	Abstract
Introduction	3	Rationale for undertaking the review	Introduction and Background
Introduction	4	Explicit review question and objectives	Introduction and Background
Methods	5	Protocol and registration status	Methodology – Design and Reporting
Methods	6	Eligibility criteria	Methodology – Design and Reporting; Table 1
Methods	7	Information sources	Information Sources and Search Strategy
Methods	8	Search strategy	Information Sources and Search Strategy; Table 2
Methods	9	Source-selection process	Study Selection and Data Charting
Methods	10	Data-charting process	Study Selection and Data Charting
Methods	11	Data items charted	Study Selection and Data Charting
Methods	12	Critical appraisal of individual sources, if performed	Methodology – Design and Reporting; not performed
Methods	13	Methods used to synthesize and present charted evidence	Synthesis
Results	14	Numbers of sources screened, assessed, excluded, and included	Search Results and Characteristics of the Evidence; Figure 1
Results	15	Characteristics of included sources	Characteristics of Included Sources; Table 3
Results	16	Critical-appraisal results, if performed	Not applicable
Results	17	Results relating to individual sources of evidence	Evidence Map; Table 4; Themes 1-7
Results	18	Synthesis of results	Themes 1–7; Figure 2; Tables 4-6
Discussion	19	Summary of principal evidence and its relevance	Discussion
Discussion	20	Limitations of the review	Limitations
Conclusions	21	Overall interpretation and implications	Conclusions
Funding	22	Sources of funding/support	Additional Information – Disclosures

**Table 8:** Data charting instrument. A piloted data-charting instrument was used to extract and organize information from the included sources. The instrument was developed specifically for this review based on the prespecified review objectives and informed by JBI guidance for scoping reviews [39]. Table Credits: Xiao Du, Nuo Wang, Abudukadier Aihemaijiang, and Lei Zhao. This data-charting instrument was developed for the present scoping review and was not reproduced from any previously published instrument. JBI: Joanna Briggs Institute.

Data Field	Information Charted
Record ID	Unique identifier assigned to the included source
Citation	Author(s), title, source, and publication details
Publication Year	Year of publication
Country or Region	Country, region, or multinational context represented
Medical Tradition	Conventional medicine, traditional medicine, integrative medicine, Indigenous/ethnomedicine, Uyghur medicine, or other relevant tradition
Setting	Hospital, health service, health system, policy/regulatory setting, or other relevant organizational context
Study/Evidence Design	Review, empirical study, audit/record review, conceptual/policy analysis, guideline, accreditation standard, or other authoritative source
Quality Domain	Healthcare quality, clinical governance, patient safety, accreditation, quality improvement, outcome measurement, or related domain
Knowledge Process	Knowledge creation, capture, appraisal, codification, preservation, sharing, translation, retrieval, application, or organizational learning
Organizational Mechanism	Governance, pathway standardization, competency development, digital infrastructure, incident learning, communication, integration, or other mechanism
Outcomes or Indicators	Reported clinical, safety, organizational, workforce, patient-experience, implementation, or process outcomes/indicators
Barriers	Reported barriers to implementation, quality improvement, or knowledge management
Facilitators	Reported facilitators or enabling conditions
Relevance to Uyghur Medicine Services	Directly relevant, immediately applicable policy/context evidence, or mechanism-based transferable evidence
Reviewer Notes	Additional information relevant to interpretation or synthesis
